# The Evaluation of Patient‐Controlled Analgesia Compared to Standard Opioid Analgesic Therapy in Pain Management Among Patients With Sickle Cell Disease: A Systematic Review and Meta‐Analysis Protocol

**DOI:** 10.1002/hsr2.70577

**Published:** 2025-03-18

**Authors:** Maria Pramila D'Costa, Melita Sheela Alva, Seyed Aria Nejadghaderi, Salha Humaid Al Bloushi, Wafa Ibrahim Saleh Al Shizawi, Nikhil Muduli, Sana Ahuja, Salma Al‐Amri, Alireza Mosavi Jarrahi, Nabeel Al‐Yateem, Syed Azizur Rahman, Amina Mohammed Al‐Marzouqi

**Affiliations:** ^1^ Oman College of Health Sciences—North Batinah Branch Suhar Sultanate of Oman; ^2^ Saskatchewan Indian Institute of Technology Saskatoon Canada; ^3^ HIV/STI Surveillance Research Center, and WHO Collaborating Center for HIV Surveillance, Institute for Futures Studies in Health Kerman University of Medical Sciences Kerman Iran; ^4^ Sikkim Manipal Institute of Medical Sciences Gangtok India; ^5^ Vardhman Mahavir Medical College and Safdarjung Hospital New Delhi India; ^6^ Department of Nursing Oman College of Health Sciences Dhofar Branch Salalah Sultanate of Oman; ^7^ Cancer Research Center Shahid Beheshti University of Medical Sciences Tehran Iran; ^8^ Department of Nursing, College of Health Sciences University of Sharjah Sharjah UAE; ^9^ Department of Health Care Management, College of Health Sciences University of Sharjah Sharjah UAE

**Keywords:** pain management, patient‐controlled analgesia, protocol, sickle cell disease, standard opioid analgesia, systematic review, vaso‐occlusive crisis

## Abstract

**Background and Aims:**

Sickle cell disease (SCD) is an inherited blood disorder characterized by the production of abnormal hemoglobin S (HbS), leading to the deformation of red blood cells into a sickle shape under low oxygen conditions. These deformed cells impede blood flow, causing vaso‐occlusive crises (VOCs), which result in severe pain, multiorgan damage, and increased mortality. Despite advancements in understanding the pathophysiology and management of VOCs, optimal pain management remains a significant challenge. This review aims to evaluate patient‐controlled analgesia compared to standard opioid analgesic therapy in pain management among patients with SCD.

**Methods:**

The relevant studies will be searched using a well‐formulated search strategy using databases such as PubMed, Embase, Scopus, and Web of Science. It will be screened by two reviewers independently (screening phase), and further, the third reviewer may solve if any discrepancies are noted. The primary outcomes will be pain reduction in intensity and frequency of breakthrough pain reported using a standard pain scale. The secondary outcomes will be adverse reactions, mortality rate, length of hospital stay, and 30‐day readmission. Then, the eligible studies are assessed for risk for bias and quality by two review members using the Newcastle–Ottawa Scale for observational studies or Cochrane Risk of Bias assessment tool version 2. Besides, it provides strong evidence in support of patient‐controlled analgesia (PCA) as an optimal pain management strategy compared to other pain management strategies. It also explores PCA's safety profiles and common adverse events to provide evidence‐based recommendations and establish a standard of care for treating SCD patients.

**Results:**

PRISMA format of reporting systematic review and meta‐analysis protocols will be followed while presenting the results of this study.

**Conclusion:**

The findings potentially influence clinical practice, healthcare policy, and future research thereby guiding the development of evidence‐based standards for VOC management in SCD patients.

**Trial Registration:**

CRD42024573178.

## 1 Introduction

Sickle cell disease (SCD) is an inherited blood condition due to the homozygosity replacing glutamic acid with valine in the β‐subunit of the hemoglobin molecule, causing abnormal hemoglobin variants (Hb S, C, D, E, and O‐Arab and β‐thalassemia) and thereof different forms of SCD [1]. HbSS is the most common and severe form of the disease compared to the other variants [1]. The long‐chain variant of hemoglobin S undergoes polymerization with an oxygen deficit, triggering red blood cells (RBCs) to form a sickle shape, diminishing their flexibility [2]. The RBCs with abnormal hemoglobin variants are quite rigid and have a shorter lifespan than normal RBCs' hemoglobins. [1] Sickled blood cells also differ from normal RBCs with the distinct feature of elevated levels of adhesion of sickled RBC molecules, causing their endothelium wall to attach and clump together, forming a blockage or impeding the blood flow in the narrow capillaries leading to vaso‐occlusive crises (VOCs), further progressing to multiorgan failure, and increased mortality [1, 2].

The mechanism of vaso‐occlusion is complex. The quick hemolysis and formation of the immature RBCs called reticulocytes as a compensatory mechanism leads to further malfunctioning of the surrounding endothelial cells [3]. As the endothelial cell damage continued, cytokine release caused monocyte leakage due to increased endothelial cell permeability. As the RBC sickling and hemolysis progress, inflammatory reactions with other blood elements, such as white blood cells and platelets, are formed [4]. The continual formation of deformed RBCs, accelerating hemolysis, trapped RBCs containing HbS adhering to the vascular endothelium, and the release of inflammatory mediators in the endothelium of the blood vessels [2] make the microcirculation block, causing cellular damage and cell death of the vital organs, leading to severe consequences like anemia and life‐threatening conditions such as acute chest syndrome, bone avascular necrosis, stroke, and pulmonary hypertension [5, 6] the most prevalent of which are periodic painful VOC [7].

A VOC is an acute painful event among patients with SCD due to an impediment in the blood flow [8, 9]. Initially, the patient's complaints are confined to fatigue and generalized body pain, which can progress severely, leading to hospitalization and the need for opioid analgesics [10]. Patients with SCD sometimes end up in the emergency department or hospitalized due to acute crises, as managing VOCs is challenging at times. Although most of these painful episodes can be managed at home, the intensity of vaso‐occlusive pain varies, with severe pain requiring significantly higher doses of medication and longer hospital stays [8, 11].

Prompt and efficient pain relief is crucial for effective pain control during VOCs. Combining oxygen, fluids, analgesics, and supportive care can alleviate this pain [12]. VOCs should be treated as an acute medical emergency, following protocols based on standard guidelines [13, 14]. They include pain assessment, providing proper analgesia within 30 min of hospital presentation, monitoring vital signs, and ruling out other causes of pain [13]. Healthcare providers should consider the type, dose, strength, and route of pain medications to manage and control painful episodes of VOCs. Studies have reported that a multimodal approach is ideal [8, 13]. However, there is a lack of standard protocol for managing pain due to VOCs that fit all age groups of patients with SCD; opioids continue to be the treatment of choice in most of the cases [15, 16]. Both The American Society of Hematology [13] (ASH) and the National Heart, Lung, and Blood Institute expert panel updated report [14] support the practice of administering parenteral opioids promptly according to a patient‐centered, SCD‐specific prescription and monitoring protocol for managing VOC pain in SCD patients.

Managing the pain of SCD is highly complex and necessitates ongoing adjustments to comfort strategies, particularly with the increasing doses of non‐opioid and opioid analgesics [17]. Frequent administration of intravenous opioids is necessary to alleviate the discomfort and possible side‐effects and life‐threatening adverse events [1]. A patient‐controlled analgesia (PCA) is one of the promising approaches [18]. PCA is a mode to self‐administer the needed doses of parenteral pain medications to manage pain control. In this method, the patient needs to push the electronic device's button to release a small quantity of opioid analgesic in case of any pain [1]. Some considerations before programming the device include physical characteristics, baseline pain level, and the analgesic used. The dose and duration between the doses are programmed according to the healthcare provider's order. The most common opioid used is morphine sulfate [19]. Patients using PCA gain control of pain symptoms as they can titrate the dose independently but within the programmed parameters [20]. Besides pain control, PCA use has been found to reduce hospital length of stay and readmission for PCA in patients with acute VOCs [17, 20]. PCA reduces anxiety developed among patients due to less medication dose and reduced frequency of breakthrough pain, which allows more patient‐focused care [1]. In addition, low morphine dosage was sufficient to have a reduced pain level in a group of patients with SCD patients when administered using PCA compared to the standard opioid therapy [21].

The possible drawbacks of utilizing PCA are mainly cooperation from the patient as it requires constant motivation from the patient side to control the pain [19]. Secondly, there can be opioid complications and the cost‐effectiveness factor as the devices can be more expensive than the standard method of treatment [19]. Some of the outcomes of the PCA are unsatisfactory as the best evidence practice regarding optimal PCA dosing is lacking [18]. This indicates a critical gap in the literature about the efficacy of PCA when compared to standard opioid therapy without PCA for managing pain in patients with SCD experiencing VOCs.

In this systematic review and meta‐analysis protocol, we used the PICO framework to define the clinical problem rigorously: P—Population: Adults and children with SCD experiencing VOCs; I—Intervention: patients receiving opioid analgesics using PCA method; C—Comparison: patients receiving standard opioid therapy without PCA. Standard opioid therapy refers to the conventional method of opioid administration for pain management in SCD patients during VOCs. Typically, this treatment involves the administration of opioids such as morphine or hydromorphone, either intravenously, orally, or via other routes, under the control of healthcare providers. This approach follows standard clinical protocols, where opioids are given on a fixed schedule or on‐demand depending on the severity of the pain; and O—Outcomes: pain reduction and frequency of breakthrough pain, opioid‐related adverse events (drowsiness, allergic reaction, lack of energy, nausea, and vomiting), mortality rates, length of hospital stay, and 30‐day readmission rates. Therefore, this systematic review and meta‐analysis protocol's primary aim is to evaluate and compare PCA with standard opioid therapy (without PCA) in pain management among patients with SCD. Furthermore, it will explore variations in PCA effectiveness across different age groups. We also aim to address the inconsistent findings in previous studies by synthesizing evidence on PCA's impact on both clinical outcomes and healthcare use, including its potential to reduce anxiety, improve patient autonomy, and optimize pain management to inform protocols for broader adoption in diverse healthcare settings.

## 2 Methods

The systematic review protocol has been prepared based on the Preferred Reporting Items for Systematic Reviews and Meta‐Analyses Protocol (PRISMA‐P) [22] (Supporting Information S1: Table S1). It has been registered in PROSPERO [Registration number: CRD42024573178].

### 2.1 Search Strategy

We will search databases such as PubMed, Embase, Scopus, Web of Science, and Google Scholar, using terms such as (“Sickle Cell Anemia,” OR “Hemoglobin S Disease,” OR “Sickle Cell Disease,”) AND (“Patient‐Controlled Analgesia,” OR “Patient Controlled Analgesia” OR “Patient Controlled Analgesic”) (Supporting Information S1: Table S2). No filters will be applied during the search for the articles. We will import all electronic search results to the EndNote X 9.0 reference manager tool. Then, the duplicates will be removed. In addition, we will endeavor to find other potential articles that may meet the criteria by doing searches of both previous and subsequent citations from the included studies in the full‐text review. We will contact the authors of articles to request the complete texts, supplementary data, and unpublished trials in case we cannot find their full text or the relevant data. The contact with the corresponding author will be performed up to three times within 1 week.

### 2.2 Eligibility Criteria

#### 2.2.1 Type of Participants

Adult and children participants diagnosed with any variant of SCD and/or VOCs will be included in the study, irrespective of their origin, language, and living country. Participants with underlying diseases and at any age are eligible. Patients with SCD should be receiving PCA as an intervention, while others with standard opioid therapy either intravenously or orally as a comparison group. The review will not include the PCA with a basal rate infusion.

#### 2.2.2 Types of Interventions

The type of medication administered through PCA may be morphine, fentanyl, hydromorphone (Dilaudid), and methadone. The included studies should have participants receiving any of the medications mentioned above through the PCA. In contrast, the standard opioid therapy, without PCA is used to achieve at least one of the primary or secondary outcomes identified for this systematic review.

#### 2.2.3 Types of Outcome Measures

##### 2.2.3.1 Primary Outcome

▪ Pain reduction in intensity and frequency of breakthrough pain will be reported using a standard pain scale.

##### 2.2.3.2 Secondary Outcomes

▪ Comparisons between the frequency of adverse events in the PCA treatment group versus the standard intervention group.
▪ Comparisons between the mortality rates among patients who received PCA treatment group compared to those who received the standard intervention group.
▪ Comparisons between the length of hospital stay among patients who received PCA treatment and who received the standard intervention group.
▪ Comparisons between the 30‐day readmission rates among patients who received PCA treatment group compared to standard intervention group.

#### 2.2.4 Types of Studies

Peer‐reviewed randomized controlled trials (RCTs) or observational studies on individuals with SCD and/or VOCs will be included. Studies will be included irrespective of their language or country.

#### 2.2.5 Exclusion Criteria

- Studies on participants with pain but without SCD.
- Studies on participants who did not receive PCA as an intervention.
- Studies on patients who received basal rate PCA.
- Studies that do not have a control group that received an intervention other than PCA.
- Studies that do not provide effect estimates in odds ratios, hazard ratios, or risk ratios or do not allow the computation of such effect sizes by providing raw data.
- Other study designs include case reports, case series, editorials, meta‐analyses, commentary letters, reviews, conference proceedings, protocols, reanalysis of previously published, and in vitro and animal studies.

### 2.3 Data Collection and Analysis

#### 2.3.1 Study Selection

Two researchers will review each article's title and abstract separately, using predetermined inclusion and exclusion criteria. Relevant studies will have their full texts found. After that, two reviewers will review the full texts separately to decide which papers meet the criteria. If there are disagreements, the reviewers will discuss and try to resolve the issue. A third‐party consultant will be approached to reach a consensus if they cannot agree. We will create an updated PRISMA flow diagram to determine the study selection process [23].

#### 2.3.2 Data Collection

The data extracted from the chosen studies will be organized using an Excel sheet (Microsoft Office). Two independent reviewers will conduct the screening process, and a third reviewer will solve any discrepancies. The extracted data will comprise the following details: first author, year, country, study design, total participants and number of participants in each group, age and sex characteristics of total participants and participants in each group, comorbidities, history of VOCs, intervention characteristics (e.g., the dose, route and schedule of PCA), the characteristics of control measures (e.g., type of modality, dose, and schedule), duration of follow‐up, race/ethnicity, and number of each primary and secondary outcomes of interest in each group.

#### 2.3.3 Study Quality Assessment

The designated studies will be appraised for bias risk and quality. Two review authors will independently appraise the studies using the Cochrane Collaboration's Risk of Bias Tool version 2 (RoB 2) for RCTs [24]. This tool assesses bias across five domains: the randomization process, deviations from proposed interventions, missing outcome data, outcome measurement, and selection of the reported result, as well as overall bias [24]. A third reviewer will be called to resolve if any discrepancies between the reviewers are observed. A summary of the quality results will be drafted and presented in a table or figure. For observational studies, we will use the Newcastle–Ottawa Scale, a recognized tool for assessing the methodological quality of observational studies [25].

#### 2.3.4 Publication Bias

If at least 10 eligible studies for each outcome, a funnel plot will be used to present the treatment effects of individual studies against the standard errors. A symmetric funnel plot indicates that it is free from publication biases [26]. Asymmetry of the funnel plot reflects heterogeneity between the studies. An Egger's test will be used for continuous and dichotomous data with no heterogeneity. A significant result of *p* < 0.05 confirms the publication bias.

### 2.4 Data Synthesis and Statistical Analysis

In this meta‐analysis, we will assess dichotomous and continuous variables to evaluate the effectiveness of the interventions under study. Dichotomous variables will be communicated as odds ratios (ORs) with 95% confidence intervals (CIs). This includes outcomes like the incidence of adverse events. For continuous variables, we will report outcomes using mean differences (MDs) or standardized mean differences (SMDs) with 95% CIs, depending on the measure's consistency across studies. This encompasses outcomes such as clinical pain scores and length of hospital stay. To address variability in the pain scales used across studies, we will standardize the pain scores, converting them into a common scale where possible. Specifically, we will aim to convert pain scores into a standard unit, such as the 0–10 numerical rating scale, using established conversion algorithms, where applicable. Additionally, we will consider the use of a statistical method known as the z‐score transformation, which has been shown to be effective for normalizing data from different pain scales to a common scale, so that the results are comparable across studies [27]. We will use STATA 16 (STATA Corp LLC, TX) software for the meta‐analysis.

Furthermore, subgroup analysis and meta‐regression will be run to explore possible sources of heterogeneity based on relevant variables like study design or study quality using the *I*² statistic derived from *Q* tests, with an *I*² value exceeding 40% suggesting significant heterogeneity [28]. If the heterogeneity exceeds this threshold, we will apply a random‐effects model; otherwise, a fixed‐effect model will be used. Moreover, we will perform a subgroup analysis to assess the outcomes for adults and children separately, as age‐related differences in treatment and response may contribute to heterogeneity in the results. We will use a continuity correction of 0.5 if the outcome of interest includes studies with zero events in one or both groups to enable their inclusion in the meta‐analysis. This approach ensures that the studies are not excluded due to zero‐event data, which could otherwise skew the results or reduce the overall statistical power. If applicable, studies with substantial variations or high heterogeneity may undergo a leave‐one‐out analysis to determine their impact on the overall results. This sensitivity analysis involves methodically omitting one study at a time from the meta‐analysis to assess the stability and robustness of the findings [28]. The original authors will be contacted in case any missing data is noticed and the analysis will be performed based on the available data.

## 3 Discussion

The scientific and technological breakthrough in the form of PCA has been promising in the efficient pain management of various medical and surgical conditions, including patients with SCD experiencing VOCs. Although multimodal approaches to pain management have emerged, there is no single protocol for pain management of VOCs in SCD that fits all age groups. Moreover, the protocols are institution‐based and based on ASH guidelines. Therefore, we believe the broader benefits of PCA in improving patient outcomes in adults and children with SCDs have been untapped. Hence, we will aim to evaluate PCA compared to standard opioid analgesic therapy in pain management among patients with SCD through a systematic review and meta‐analysis.

The effectiveness of PCA in managing pain and other patient outcomes is compared to the standard opioid therapy using intravenous or oral, including the nonpharmacological treatments used for the VOC among SCD patients. The main findings of the systematic review and the meta‐analysis will be the summary and interpretation done in the context of the aim and the objectives aligned with the primary and secondary outcomes of the SCD patients. Our review aims to contribute to developing standardized, evidence‐based protocols for PCA use, which is particularly important given the challenges and variability in managing pain among SCD patients with VOCs. This systematic review and meta‐analysis could significantly impact clinical practice, patient outcomes, healthcare policy, standards and protocols, and future research directions in the pain management of SCD. PCA permits the client to have control over the VOC within the set limits, allowing them to tailor the pain medication to the needs of the patient. Therefore, this study could be clinical evidence on whether PCA can be an optimum pain management strategy for adults and children with SCD compared to standard opioid therapy.

Furthermore, efficient pain control may improve patient satisfaction, quality of life, duration of hospital stay, and readmission. In addition, this study provides insights into the PCA's safety profiles, including common adverse events. This results will be disseminated through a peer‐reviewed journal.

## Author Contributions

**Maria Pramila D'Costa:** conceptualization, writing – review and editing, writing – original draft, methodology, validation, investigation, software, resources. **Melita Sheela Alva:** writing – original draft, writing – review and editing. **Seyed Aria Nejadghaderi:** writing – original draft, writing – review and editing, supervision, project administration, investigation, conceptualization, methodology. **Salha Humaid Al Bloushi:** writing – original draft, writing – review and editing. **Wafa Ibrahim Saleh Al Shizawi:** writing – review and editing, writing – original draft. **Nikhil Muduli:** writing – original draft, writing – review and editing. **Sana Ahuja:** writing – review and editing, writing – original draft. **Salma Al‐Amri:** writing – original draft, writing – review and editing. **Alireza Mosavi Jarrahi:** writing – review and editing, writing – original draft. **Nabeel Al‐Yateem:** writing – original draft, writing – review and editing. **Syed Azizur Rahman:** writing – review and editing, writing – original draft. **Amina Mohammed Al‐Marzouqi:** writing – original draft, writing – review and editing.

## Ethics Statement

Ethical approval is not required as it is a protocol. However, the study results will be disseminated through peer‐reviewed journals and presented at the conferences.

## Conflicts of Interest

The authors declare no conflicts of interest.

## Transparency Statement

The lead authors Maria Pramila D'Costa and Seyed Aria Nejadghaderi affirm that this manuscript is an honest, accurate, and transparent account of the study being reported; that no important aspects of the study have been omitted; and that any discrepancies from the study as planned (and, if relevant, registered) have been explained.

## Supporting information

Supporting information.

## Data Availability

The authors have nothing to report.
